# Microstructural evolution of tantalum nitride thin films synthesized by inductively coupled plasma sputtering

**DOI:** 10.1186/s42649-020-00026-7

**Published:** 2020-02-27

**Authors:** Sung-Il Baik, Young-Woon Kim

**Affiliations:** 1 Research Institute of Advanced Materials, Department of Materials Science and Engineering, Seoul National University, 1 Gwanak-ro Gwanak-gu, Seoul, Republic of Korea; 2 Present address: Department of Materials Science & Engineering, Northwestern University, Evanston, IL 60208 USA

**Keywords:** Tantalum nitride (TaN), Inductively coupled plasma (ICP), Transition metals, Thin film, Microstructure, Transmission electron microscopy (TEM)

## Abstract

Tantalum nitride (TaN_x_) thin films were grown utilizing an inductively coupled plasma (ICP) assisted direct current (DC) sputtering, and 20–100% improved microhardness values were obtained. The detailed microstructural changes of the TaN_x_ films were characterized utilizing transmission electron microscopy (TEM), as a function of nitrogen gas fraction and ICP power. As nitrogen gas fraction increases from 0.05 to 0.15, the TaN_x_ phase evolves from body-centered-cubic (b.c.c.) TaN_0.1_, to face-centered-cubic (f.c.c.) δ-TaN, to hexagonal-close-packing (h.c.p.) ε-TaN phase. By increasing ICP power from 100 W to 400 W, the f.c.c. δ- TaN phase becomes the main phase in all nitrogen fractions investigated. The higher ICP power enhances the mobility of Ta and N ions, which stabilizes the δ-TaN phase like a high-temperature regime and removes the micro-voids between the columnar grains in the TaN_x_ film. The dense δ-TaN structure with reduced columnar grains and micro-voids increases the strength of the TaN_x_ film.

## Introduction

Tantalum nitride (TaN_x_) has been widely used for wear resistance coatings, diffusion barrier layers, and high-density magnetic recording media because of its good mechanical properties, chemical inertness, wide band-gap, and high-temperature stability (Choi and Yoon 2004; Han et al. 1998; Laurila et al. 2001; Li et al. 1997). Unlike the most common metal nitride, TaN_x_ has a complex system with many equilibrium phases, i.e., amorphous, body-centered-cubic (b.c.c.) α-Ta(N_0.1_), hexagonal-close-packing (h.c.p.) γ-Ta_2_N, face-centered-cubic (f.c.c.) δ-TaN, h.c.p. ε-TaN, and meta-stable phases with defects (Kawasaki et al. 2001; Stavrev et al. 1997; Wiesenberger et al. 1988). Due to the complexity of the Ta-N system, its microstructural change is very sensitive to the deposition method and growth conditions. Various techniques have been adopted to obtain high-quality TaN_x_ thin films, such as reactive sputtering (RSP) (Lee et al. 2001; Riekkinen et al. 2002), chemical vapor deposition (CVD) (Cho et al. 1999; Park et al. 2002), and ion beam deposition (IBED) (Baba and Hatada 1996; Ensinger et al. 1995). In these techniques, the major concerns for minimizing the porous metastable microstructure have still not been resolved, which is critically related to the degradation of TaN_x_ film’s mechanical and electrical properties (Ohring 1992).

Inductively coupled plasma (ICP) assisted direct current (DC) sputtering has been adopted as a promising technique to develop a higher density of plasma at a lower deposition temperature (Lee and Joo 2003; Rossnagel and Hopwood 1993; Lee et al. 2005). ICP is known to be able to generate an ion density up to 100 times higher than normal DC and capacitively coupled radiofrequency (RF) plasma (Hopwood and Qian 1995). Due to the high density of ion, it is reported to form TaN_x_ thin films with good mechanical and electrical properties (Lim et al. 2000; Sreenivasan et al. 2007; Baik et al. 2008), which is mainly controlled by the internal structure of void formation (Shin et al. 2002a), preferred orientation (Shin et al. 2002b), and phases formed (Kim and Cha 2005).

In the present study, we investigate the mechanical behavior and microstructural evolution of TaN_x_ films grown at a temperature of 100 °C with changes of nitrogen fraction and ICP power. Based on the observation of microstructural evolutions utilizing transmission electron microscopy (TEM), formation sequences and phase distributions for TaN_x_ thin film are proposed.

## Experimental details

The growth of TaN_x_ films was performed by the DC magnetron sputtering system combined with high density inductively coupled plasma (ICP) using external antenna (Lee et al. 2007). DC bias was fixed at 150 W, and the nitrogen gas fraction (***f***_*N2*_), N_2_/(Ar + N_2_), changed from 0.05 to 0.15 while ICP power was set in the range of 100-400 W. Deposition chamber pressure was maintained at 20 mTorr (2.67 Pa) during the growth. The substrate temperature was 100 °C during the growth until 1 μm thickness of TaN_x_ film. The microhardness of the films was measured by averaging 8 points using a Hisherscope H100CXYp instrument with a load of 20mN.

Plan-view and cross-sectional transmission electron microscopy (PTEM and XTEM, respectively) samples were prepared by grinding, and dimpling followed by 4 kV Ar^+^ ion milling utilizing a Gatan precision ion polishing system (PIPS). TEM observations were performed utilizing the FEI F20 and JEOL JEM-3000F, operated at 200 and 300 kV, respectively. Selective area diffraction patterns (SADPs) were indexed using the Crystal-Maker and Crystal-Diffract programs (Palmer and Palmer 1994).

## Results and discussion

**Fig. 1:**
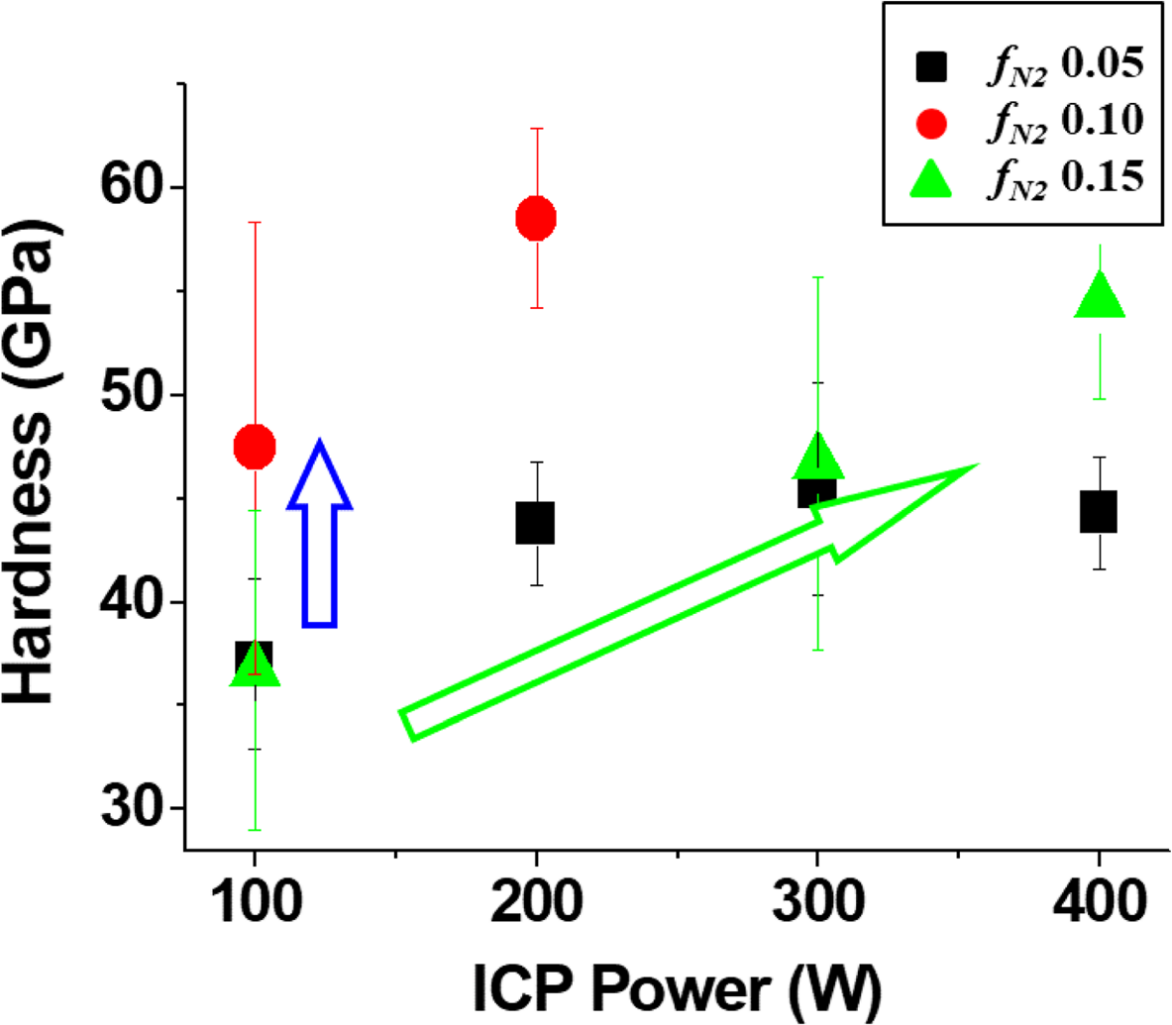
Microhardness of TaN_x_ films deposited by ICP sputtering, where ICP power was varied from 100 to 400 W at three nitrogen gas fractions (***f***_*N2*_), 0.05 (black squares), 0.1 (red circles), and 0.15 (green triangles)

The effects of ICP power and nitrogen gas fraction (***f***_*N2*_) on the mechanical property of TaN_x_ films were measured by microhardness, Fig. 1. The microhardness value with ICP sputtering is in the range of 36–60 GPa, which is 20–100% higher than that of the conventional sputtering method, ~30GPa (Shin et al. 2002b; Lee et al. 2005). Even though some data points are missing due to the brittle structure of TaN_x_ films, the trend of microhardness can be found, increasing as ICP power increases, but not proportional to the increase in the nitrogen gas fraction (***f***_*N2*_). The microhardness of TaN_x_ film with 0.1 ***f***_*N2*_ has the highest value among the three ***f***_*N2*_ conditions, 0.05 (black squares), 0.1 (red circles), and 0.15 (green triangles). The microhardness of TaN_x_ film increases up to ~60 GPa with 0.1 ***f***_*N2*_ and 200 W ICP power. The microhardness values with 0.15 ***f***_*N2*_ have similar values with those at 0.05 (black squares), 36–47 GPa, between 100 and 300 W ICP power (green triangles), but slightly higher value at 400 W, 54 vs. 43 GPa. The increase of hardness with ICP power is well matched with the previous microhardness results (Lee et al. 2007).

**Fig. 2:**
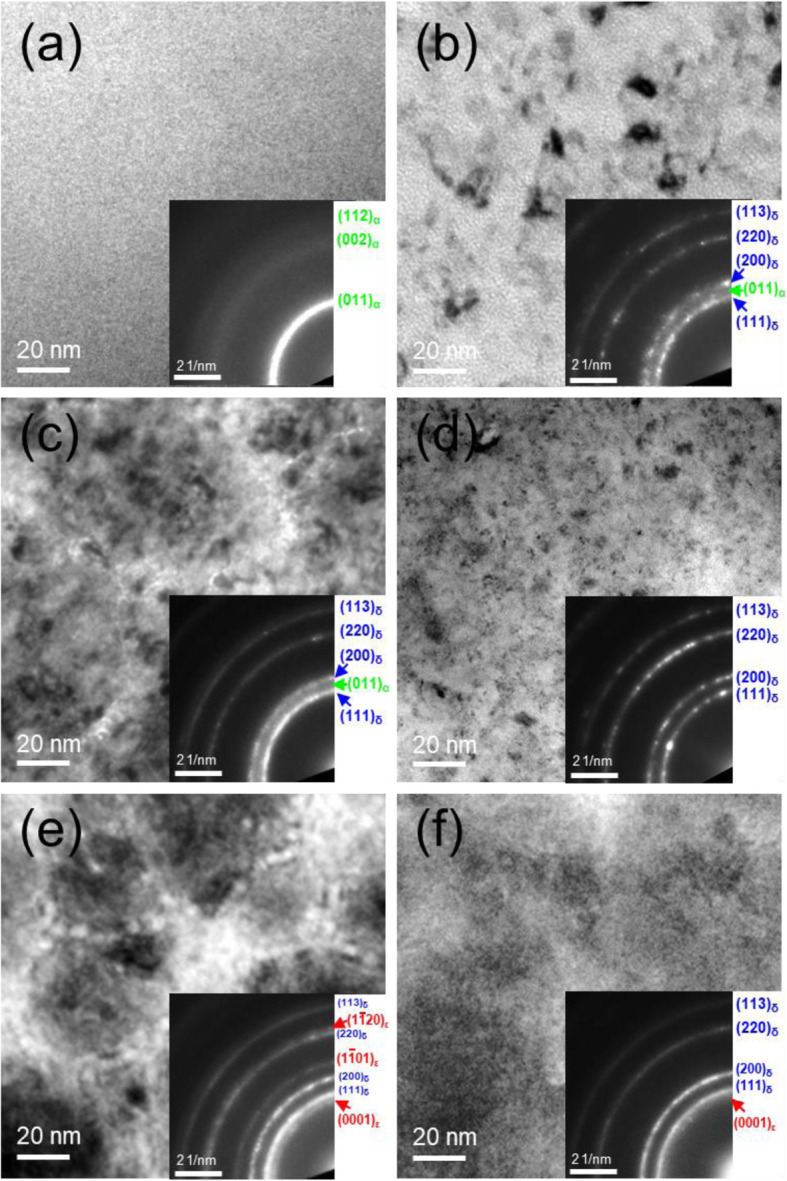
Bright-field (BF) plan-view (P) TEM and selective area diffraction pattern (SADP) analyses of the top region of TaN_x_ films grown with different nitrogen gas fractions (***f***_*N2*_) and ICP powers (W). (a, b) 0.05 ***f***_*N2*_ at 100 W and 400 W, (c, d) 0.1 ***f***_*N2*_ at 100 W and 400 W, (e, f) 0.15% ***f***_*N2*_ at 100 W and 400 W

**Fig. 3:**
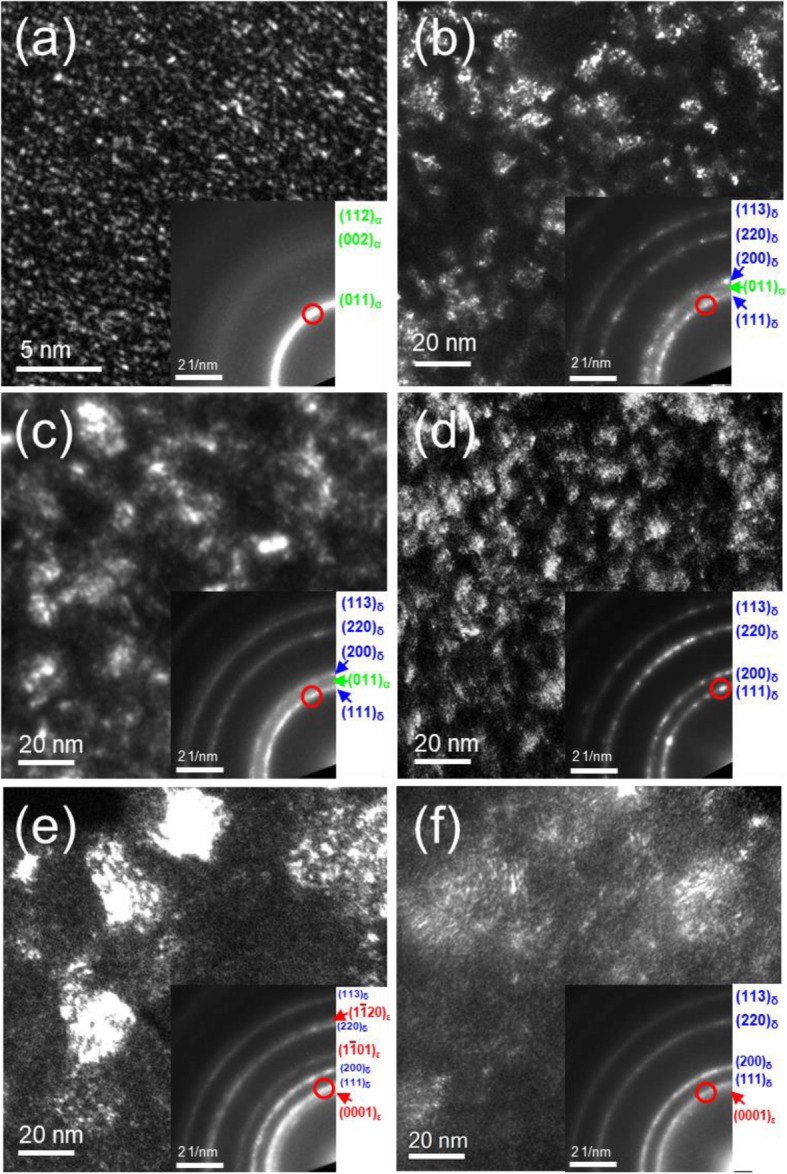
DF PTEM analyses of the top region of TaN_x_ films grown with different nitrogen gas fractions (***f***_*N2*_) and ICP powers (W). (a, b) 0.05 ***f***_*N2*_ at 100 W and 400 W, (c, d) 0.1 ***f***_*N2*_ at 100 W and 400 W, (e, f) 0.15% ***f***_*N2*_ at 100 W and 400 W. The position of DF reflection is represented by the red circle in the SADP in the inset of each image. The magnification of DF image for 0.05 ***f***_*N2*_ at 100 W in (a) is different from others to see the small precipitate

Figure 2 displays bright-field (BF) PTEM analysis of TaN_x_ films to see the microstructural evolutions in the three ***f***_*N2*_ of 0.05, 0.1, and 0.15 at the lowest and highest ICP powers, 100 W and 400 W, respectively. SADPs in the inset of BF TEM images display the phase change of TaN_x_ films as a function of ***f***_*N2*_ and ICP power. When ***f***_*N2*_ is lowered to 0.05 with ICP power at 100 W, Fig. 2a, BF TEM image displays a homogenous b.c.c. α-Ta(N_0.1_) phase with a fine grain size of 1–2 nm. The grain size is measured by dark-field (DF) TEM image in Fig. 3. The strong continues ring pattern in the SADP is indexed as (011)_α_ planes of b.c.c α-Ta(N_0.1_) phase (green color). However, the diffused ring pattern of α-Ta(N_0.1_) phase in the SADP can be explained as a very short range ordering phase or amorphous phase. As ***f***_*N2*_ increases to 0.1, Fig. 2c, f.c.c. δ-TaN becomes a major phase, and the grain size increases to 10–15 nm. And the columnar structure is weakly developed with a size of 100–150 nm. The SADP shows clear f.c.c. ring patterns of δ-TaN phase, indicated in blue, due to its larger grain size. However, the diffused ring-pattern of α-Ta(N_0.1_) phase is still observed between (111)_δ_ and (200)_δ_ planes of the δ-TaN phase, explaining the mixture of δ-TaN phase with the α-Ta(N_0.1_) phase. As ***f***_*N2*_ increases to 0.15, Fig. 2e, the h.c.p. ε-TaN is observed with the δ-TaN phase. The (0001), (1 $\overline{1}$ 01), and (11 $\overline{2}$ 0) planes of ε-TaN phase, indicated in red, are visible in the SADP analysis. The columnar structure is fully developed with the size of 100–150 nm, and δ-TaN subgrains with a size of 30–40 nm are observed within the columnar structure. When increasing ICP power from 100 to 400 W, the δ-TaN phase becomes a dominant phase in all three ***f***_*N*_, Fig. 2(b,d,f). When ICP power increases to 400 W at 0.05 ***f***_*N2*_, Fig. 2b, the δ-TaN is developed within the nanocrystalline α-Ta(N_0.1_) or amorphous phase similarly to that at 0.1 ***f***_*N2*_ and 100 W; however, the density of δ-TaN grain is lower than that in the 0.1 ***f***_*N2*_ and 100 W. In the 0.1 and 0.15 ***f***_*N2*_ at 400 W, Fig. 2(d,f), the δ-TaN grains are slightly finer and evenly distributed, and the columnar structure is nearly disappeared. Even though the δ-TaN phase was reported as a stable phase at a temperature higher than 1800 °C (Frisk 1998), it is well developed at a low substrate temperature of 100 °C during ICP assisted sputtering. The higher ICP power increases the intermixing of Ta and N species in the TaN_x_ phase and promotes the δ-TaN phase in the films.

Figure 3 displays DF PTEM analysis of TaN_x_ films to see the grain size and the phase distribution in the three ***f***_*N2*_ of 0.05, 0.1, and 0.15 at the lowest and highest ICP powers, 100 W and 400 W, respectively. The position of DF reflections is represented by the red circles in the SADP. In the 0.05 ***f***_*N2*_ and 100 W ICP power, Fig. 3a, the grain of the b.c.c. α-Ta(N_0.1_) phase has a size of 1–2 nm, which is homogenously distributed in the film. These small grain size and homogenously distribution are also represented in strong continues circles in the SADP. When increasing the ICP power to 400 W, the δ-TaN phase has developed with a size of 10–15 nm based on the α-Ta(N_0.1_) phase. As ***f***_*N2*_ increases to 0.1 with the ICP power at 100 W, Fig. 3c, the f.c.c. δ-TaN becomes a major phase with the grain size of 10–15 nm, which is highly interconnected with each other. As shown in SADP analysis above, α-Ta(N_0.1_) phase is still presented as a mixture with the δ-TaN phase. This is similar to the (α+δ) TaN mixture phase in the 0.05 ***f***_*N2*_ and 100 W ICP power; however, the density of δ-TaN grain in the DF image is higher than that in the 0.05 ***f***_*N2*_ and 400 W. In the ICP power of 400 W, Fig. 3d, the δ-TaN grain is divided more finely to the size of 6–12 nm. As ***f***_*N2*_ increases to 0.15 at 100 W, Fig. 3e, the δ-TaN and ε -TaN grain with the size of 30–40 nm are developed within the columnar structure. The higher nitrogen gas fraction may promote the mobility of Ta species to form a larger grain with the δ-TaN and ε-TaN phases. The distributions of the δ-TaN and ε-TaN phase within the columnar structure will be represented in Fig. 5b. When increasing ICP power from 100 to 400 W, Fig. 3f, the ε-TaN and the columnar structure are nearly disappeared in the TaN films. The δ-TaN phase is divided into the finer grain size but forms a group within the columnar structure as increasing the ICP power to 400 W.

**Fig. 4:**
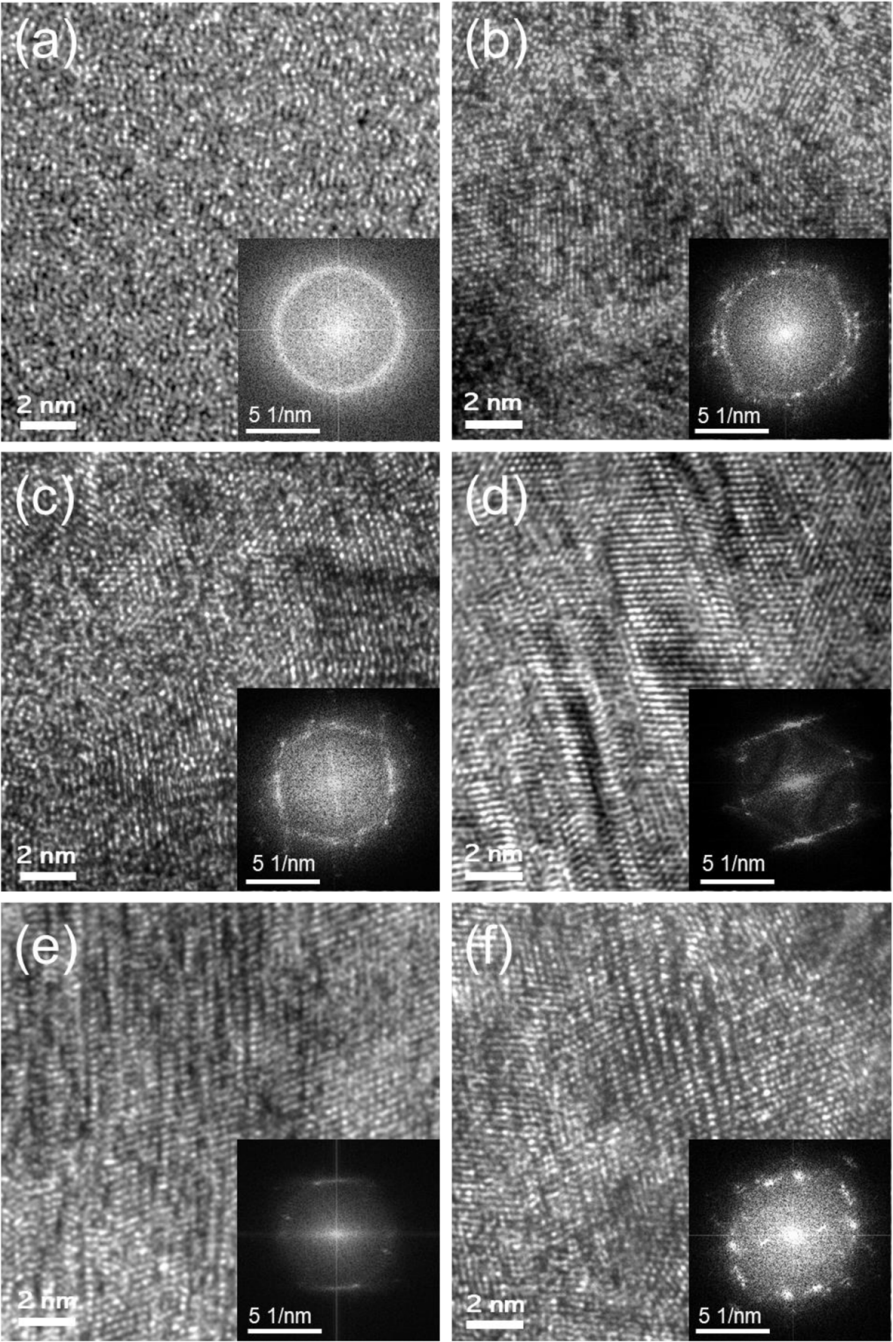
High resolution (HR) TEM analysis of the top region of TaN_x_ films grown with different nitrogen gas fractions (***f***_*N2*_) and ICP powers (W). (a, b) 0.05 ***f***_*N2*_ at 100 W and 400 W, (c, d) 0.1 *f*_*N2*_ at 100 W and 400 W, (e, f) 0.15 ***f***_*N2*_ at 100 W and 400 W

Figure 4 displays high-resolution (HR) TEM image to see the atomic structure change of TaN_x_ films grown with three ***f***_*N2*_ of 0.05, 0.1, and 0.15 at the lowest and highest ICP powers, 100 W and 400 W, respectively. The crystallographic information of each condition is also well represented by the fast Fourier transformation (FFT) in the inset. The HR TEM image in the 0.05 ***f***_*N2*_ and 100 W ICP power, Fig. 4a, shows a randomly distributed nanocrystalline α-Ta(N_0.1_) phase with size less than 2 nm. The size of α-Ta(N_0.1_) phase is very small, therefore, the phase is nearly similar to an amorphous. The structure and size of α-TaN_0.1_ phase are also well represented by the ring pattern in the FFT inset. When ICP power is increased to 400 W, Fig. 4b, the amorphous or randomly oriented nanocrystalline α-Ta(N_0.1_) phase are partially replaced by δ-TaN crystalline structure with a clear lattice image in HR analysis. In the BF image in Fig. 2b, the size of grain can be increased up to 10–15 nm; however, in this HR image, most grains are still small with a size of 4–7 nm and are disconnected by the amorphous layers. As the ***f***_*N2*_ increase to 0.1 and 0.15 at 100 W ICP power, highly faulted or composite type of δ-TaN phase is developed. The faulted structure is well represented as the streaks in the FFT analysis. When increasing ICP power to 400 W, the δ-TaN phase is still the main phase, but the atomic structure is much more complex with highly faulted structure. The composite structure of multilayer can increase the strength significantly (Baik et al. 2016).

**Fig. 5:**
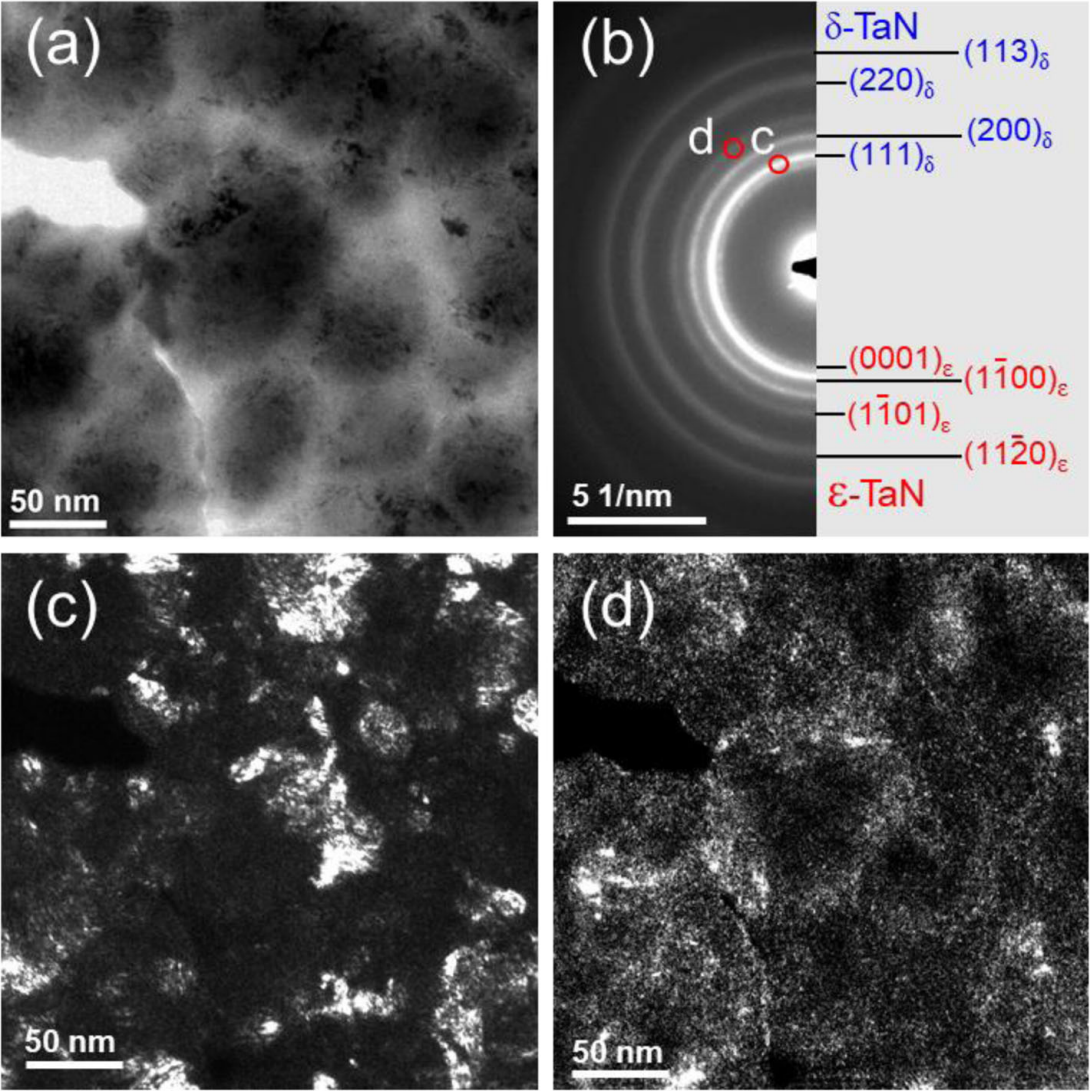
PTEM images of TaN_x_ film grown with 0.15 ***f***_*N2*_ for 100 W ICP power. (a) BF TEM image, (b) SADP and its indexing, (c, d) Dark-field (DF) TEM images of δ - TaN (111), and ε- TaN $\left(11\overline{2}1\right)$ reflections in TaN_x_ film

As the ***f***_*N2*_ increases from 0.05 to 0.1 at the 100 W ICP power, the microhardness of TaN_x_ film increases by the formation of the highly faulted or composite type of f.c.c. δ-TaN phase. However, the microhardness decreases with a formation of ε-TaN phase as the ***f***_*N2*_ increases from 0.1 to 0.15 at the 100 W ICP power. Therefore, the growth distributions of ε-TaN within the δ-TaN phase can be an important factor to explain the microhardness decrease. Figure 5 displays the distribution of δ- and ε-TaN in the films grown with at 0.15 ***f***_*N2*_ and 100 W ICP power. The detailed crystallographic information of δ-TaN and ε-TaN is well represented in the SADP analysis, Fig. 5b, and the distributions of each phase is displayed by dark-field (DF) PTEM analyses in Fig. 5 (c, d) using the (111) reflection of δ-TaN and $\left(1\overline{1}01\right)$ reflection of ε-TaN, where are represented as red circles in the SADP in Fig. 5b. The BF PTEM image, Fig. 5a, displays the top surface of the columnar structure whose boundaries appeared as lighter contrast compared to the center of the columnar grain. DF images, Fig. 5(c, d), reveal the difference of distribution for δ-TaN and ε-TaN phases; δ-TaN is mainly found at the center of the columnar grain, while ε-TaN appears at the boundaries. However, the ICP power is increased from 100 to 400 W, ε-TaN phase is disappeared by the breaking of columnar structure, as shown in Fig. 2f.

**Fig. 6:**
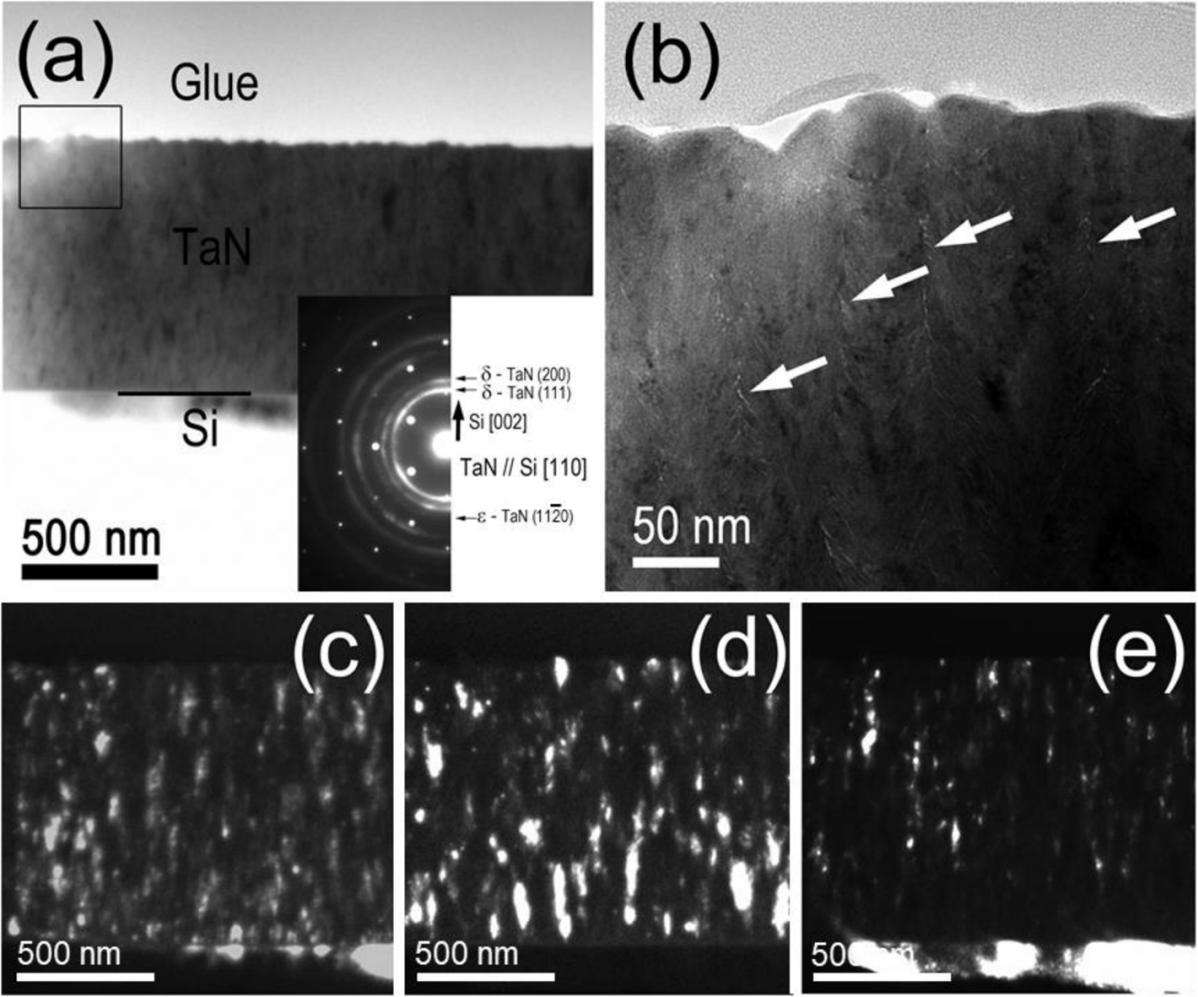
Cross-sectional **(**X) TEM analysis of TaN_x_ film grown with 0.15 ***f***_*N2*_ for 100 W ICP power. (a) BF TEM image with corresponding SADP in the inset. DF TEM images of (c) δ- TaN (111), (d) δ- TaN (200), and (e) ε- TaN $\left(11\overline{2}1\right)$ reflections in TaN_x_ film

Figure 6 is a cross-sectional (X) TEM analysis of TaN_x_ film grown with 0.15 ***f***_*N2*_ for 100 W ICP power. The distributions of δ and ε-TaN phases are observed by DF images using the reflections of (111) and (200) for δ-TaN, and $\left(1\overline{1}01\right)$ for ε-TaN, Fig. 6(c-e). The reflections for DF TEM images are represented in the SADP analysis in the inset of Fig. 6a. Each phase in the SADP is indexed on the right side of the image. Dense and small-sized columnar grains of δ-TaN are nucleated at the bottom of the films with a diameter of 50–100 nm, Fig. 6c. The (111) preferred orientation of δ-TaN phase is dominant at the initial stage of film growth; however, it is mixed with (200) orientation with an increase of columnar grain to 100–200 nm, Fig. 6(c,d). The δ-TaN phase has a strong elastic anisotropy, resulting in a strong dependency on preferred orientation (Sundgren and Hentzell 1986). It is known that (002) preferred orientation is fully grown with small compressive stress as N_2_ ion-to-metal flux ratio increases (Shin et al. 2002b). Micro-voids are formed at the columnar grain boundary as indicated white arrows in Fig. 6b. These micro-voids are observed with higher nitrogen gas fractions in the transition metal nitride and degrade the microhardness significantly (Lim et al. 2000). The formation of the ε-TaN phase is relating to the micro-voids with the higher nitrogen gas fraction around the columnar grain boundary. This micro-micro-void structure and ε-TaN phase can be reduced by the increase of ICP power due to its increased population and bombardment effect of ions during film deposition.

**Fig. 7:**
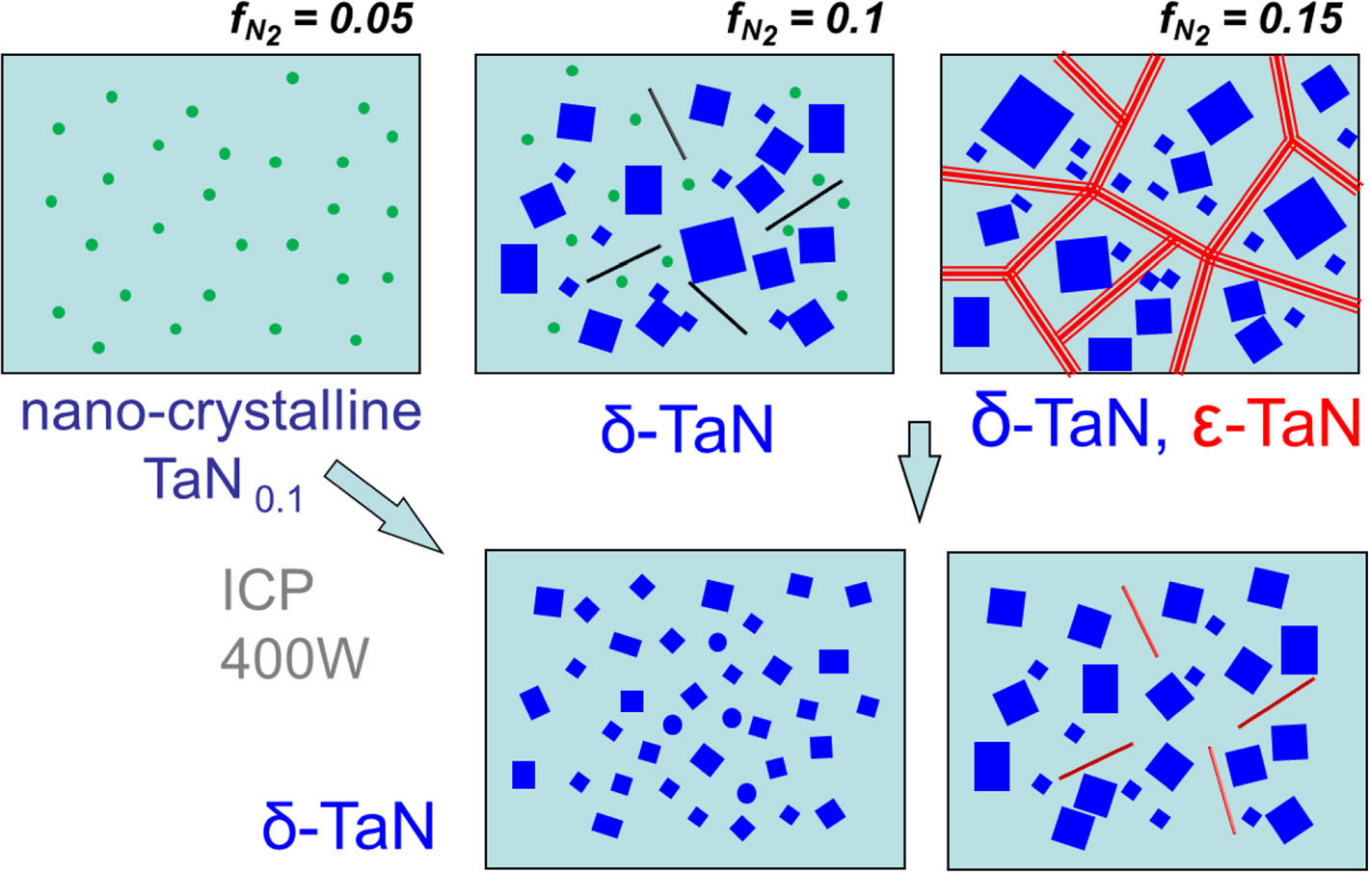
Schematic representation illustrating microstructural evolution of TaN_x_ films with the change of nitrogen gas fractions (***f***_*N2*_) and ICP power

Figure 7 is a schematic representation of the microstructural evolution of TaN_x_ films with the change of nitrogen gas fraction (***f***_*N2*_) and ICP power. As ***f***_*N2*_ increases, the phase of TaN_x_ film changes from b.c.c. α-Ta(N_0.1_), to f.c.c. δ-TaN, and to h.c.p. ε-TaN phase. These phase evolutions are accompanied by the morphology change from homogenous α-Ta(N_0.1_) nanocrystalline or amorphous film to δ and ε-TaN columnar structure. The δ-TaN grain is developed as a subgrain structure within the columnar structure. The columnar structure is weakly formed without ε-TaN phase 0.10 ***f***_*N2*_ and 100 W ICP power and gradually developed as ***f***_*N2*_ increases. The formation of the ε-TaN phase is related to the higher nitrogen gas fraction around the columnar grain boundary. However, these micro-voids and ε-TaN phase can be reduced with the increase of ICP power due to the increased population and bombardment of ions. The higher ICP power has a high capacity and high flux ion generation which can promote the intermixing of Ta and N ions during the growth of TaN_x_ film. In the phase diagram of the Ta-N system (Frisk 1998), the δ-TaN is stable at a high temperature above 1800 °C; however, the δ-TaN phase is widely grown at a low substrate temperature of 100 °C with ICP sputtering. This result is different from the previous TaN_x_ films grown at 300 °C using DC magnetron sputtering, which contains very complex phases of γ, δ, and ε-TaN phases (Lee et al. 2007). The increased mobility of Ta and N ions by ICP sputtering is able to stabilize δ-TaN phase like a high-temperature regime and reduces the micro-voids and ε-TaN phase in the TaN_x_ films. The dense δ-TaN structure with reduced columnar grains and micro-voids in-between increases the strength of the TaN_x_ film.

## Conclusions

Inductively coupled plasma (ICP) assisted DC sputtering has been used as a promising technique to improve the hardness of the TaN_x_ thin film. We investigated the mechanical behavior and microstructural evolution of TaN_x_ films as a function of nitrogen gas fraction (***f***_*N2*_) and ICP power. The following conclusions are drawn as follow;

- The microhardness value with ICP sputtering is in the range of 36–60 GPa, which is 20–100% higher than that of conventional sputtering method ~ 30 GPa. As ICP power increases, the microhardness of TaN_x_ films increases but not proportionally to the increase in the nitrogen gas fraction.
- As nitrogen gas fraction increases, the TaN_x_ phase evolves from b.c.c. α-Ta(N_0.1_), to f.c.c. δ-TaN, to the h.c.p. ε-TaN phase. These phase evolutions are accompanied by the structural changes from homogenous α-Ta(N_0.1_) nanocrystalline or amorphous film to δ and ε-TaN columnar structures.
- When increasing the ICP power from 100 W to 400 W, the δ-TaN phase becomes the main phase in all nitrogen fractions investigated. The morphology of the nanocrystalline α-Ta(N_0.1_) or amorphous phase at 0.05 ***f***_*N2*_ and 100 W also changes similarly to that of the δ-TaN phase at 0.1 ***f***_*N2*_ and 100 W.
- The columnar structure is weakly formed without the ε-TaN phase at 0.10 ***f***_*N2*_ and 100 W ICP power; however, it is gradually developed as an increase of N_2_ gas fraction. The formation of the ε-TaN phase at 0.15 *f*_*N2*_ and 100W is due to the higher nitrogen gas fraction around the columnar boundary. These columnar structures are nearly disappeared when ICP power is increased to 400 W.
- The higher ICP power stabilizes the δ-TaN phase like a high-temperature regime by enhancing the mobility of Ta and N ions and removes the micro-voids between the columnar grains in the TaN_x_ film. The dense δ-TaN structure with reduced columnar grains and micro-voids increases the strength of the TaN_x_ film.

## Data Availability

The data and the materials in the current manuscript cannot be shared because they also constitute a few on-going works, and the authors do not have the right to open them.
